# Complication rates following open surgical removal of osteosynthesis material from the pelvis and acetabulum: a retrospective case series of 154 removals

**DOI:** 10.2340/17453674.2026.45294

**Published:** 2026-01-23

**Authors:** Thore C SCHERFF, Nico HINZ, Cornelius GRIMME, Karl-Heinz FROSCH, Maximilian HARTEL

**Affiliations:** 1 Department of Trauma Surgery, Orthopedics and Sports Traumatology, BG Klinikum Hamburg, Hamburg; 2 Department of Septic Trauma Surgery and Orthopedics, BG Klinikum Hamburg, Hamburg; 3 Department of Trauma and Orthopedic Surgery, University Medical Center Hamburg-Eppendorf, Hamburg, Germany

## Abstract

**Background and purpose:**

While complication rates for primary pelvic and acetabular fracture surgeries are well documented, limited data exists on complications following osteosynthesis implant removals. We aimed to evaluate the complication rates of pelvic implant removals with respect to the surgical approach, type of implant, and indication for removal.

**Methods:**

This retrospective, consecutive case series included all patients undergoing pelvic implant removal between January 2013 and December 2023 using Kocher-Langenbeck, modified Stoppa (AIP), or ilioinguinal approaches for the removal. Isolated minimally invasive, percutaneous implant removals were excluded.

**Results:**

154 implant removals in 141 patients were analyzed. Overall complication rate was 34% (n = 53). Most common complications were intraoperative bleeding requiring transfusion (n = 17; 11%), postoperative anemia requiring transfusion (n = 12; 7.8%), and vascular injuries (n = 9; 5.8%). The ilioinguinal approach showed a higher complication rate (19/37; 51%) than the Kocher-Langenbeck (21/68; 31%) or the Stoppa/AIP approach (13/49; 27%). Removal of implants from the anterior pelvic ring and acetabulum (22/45; 49%) also had a higher complication risk than from the posterior pelvic ring and acetabulum (20/67; 30%) or of symphyseal plates (11/42; 26%). Removal due to infection also showed a particularly high complication rate (25/57; 44%) compared with aseptic indications, e.g., interfering material or removal for THA.

**Conclusion:**

Pelvic implant removals, especially from the anterior pelvic ring or acetabulum, using the ilioinguinal approach, and in case of infection, are associated with a particularly high complication risk. These findings can support clinical decision-making and informing patients on the potential risks of hardware removals.

Despite advances in techniques and implants, primary stabilization of pelvic ring and acetabular fractures carries a complication risk of about 14–41%, mostly depending on the surgical approach [1-3]. Typical complications are perioperative bleeding through vascular damage (6%), nerve injuries (12–16%), surgical site infections (SSIs) (9%), and thromboembolic events (6%) [4-6].

While complication risks associated with the primary fracture treatment are well known, data on complications of pelvic implant removal is limited. Implant removal in general can be challenging, e.g., due to the altered anatomy, scar tissue, or due to heterotopic ossifications [7,8]. Thus, implant removal may be associated with a higher complication rate compared with the primary stabilization of the pelvis.

A clear indication for implant removal from the pelvis may be given in the case of peri-implant infections, malpositioned implants, or in select cases with consolidated fractures in the developing skeleton [9-11]. In addition, in cases of residual complaints after severe trauma, patients might ask for elective hardware removal, which is why there is a need to know the potential risks. We aimed to assess complication risks of open pelvic implant removals based on the surgical approach, implant type, and indication.

## Methods

### Study design

This is a retrospective, single-center analytical case series, which has been reported in line with the PROCESS 2023 guidelines [12].

### Patients and characteristics of study center

The study was conducted at a German referral center with a specialty in aseptic and septic revision surgery in trauma and orthopedic reconstructive surgery. For a better interpretation the study center performed about 70 to 120 primary pelvic ring and acetabular fracture fixations per year during the study period. As the study center is specialized in aseptic and septic revision surgery for pelvic fractures and receives frequent referrals of patients, particularly from the northern half of Germany but also from other parts of Germany and Europe, the study population also includes several cases involving highly complex aseptic and septic revision surgeries.

All patients who underwent surgical removal of osteosynthesis implants from the pelvis between January 2013 and December 2023 were consecutively included. Inclusion criteria were implant removal using open surgery from the pelvis via 1 or more of the following surgical approaches: ilioinguinal approach, modified Stoppa/anterior intrapelvic (AIP) approach, or Kocher-Langenbeck approach.

**Figure 1:**
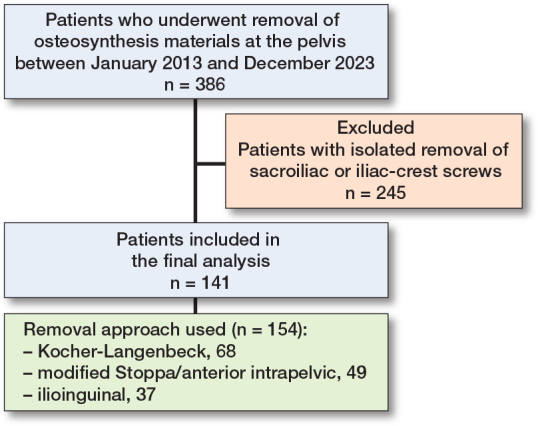
Flowchart of the patient selection process.

Patients who exclusively underwent removal of iliosacral screws or removal of plates and screws from the iliac crest were excluded, as these are usually less invasive procedures, which are associated with a distinctly different complication profile compared with the aforementioned approaches. Patients were retrospectively identified by their digital patient file based on standardized operation and procedure coding (Germany OPS-codes 5-787.0d, 5-787.1d, 5-787.2d, 5-787.3d, 5-787.kd). If more than 1 of the above-mentioned approaches was used to remove the implants in a patient (e.g., 1 anterior and 1 posterior approach), each procedure associated with 1 approach was counted as a separate event. For patient selection process see Figure 1.

### Data sources and collection

After identifying the patients, the following data was collected from the digital patient files: Age, sex, documented indication for implant removal (infection, interfering material/planned removal, removal for one-stage THA, pseudarthrosis/re-osteosynthesis), localization and type of implant removed (symphyseal plate, anterior pelvic ring/acetabulum, posterior pelvic ring/acetabulum), and surgical approach used for removal (Kocher-Langenbeck approach, modified Stoppa/AIP approach, ilioinguinal approach). One-stage THA includes all patients who underwent material removal and THA in a single operation. In patients who underwent two-stage THA (2 subsequent operations) only the 1st operation for implant removal was included and was assigned either to the group of infections or to the group of planned removal.

### Definition of complications

Any revision surgery due to complication and the following perioperative in-hospital complications during the index hospital stay were then collected from the patient records: intraoperative bleeding requiring transfusion, hematoma requiring revision surgery, vascular injury, postoperative infection, nerve damage/disorder of the femoral nerve, nerve damage/disorder of the lateral femoral cutaneous nerve (LFCN), nerve damage/disorder of the sciatic nerve, intraoperative urinary bladder injury, deep vein thrombosis (DVT), wound healing disorder, postoperative anemia requiring transfusion, and intraoperative myocardial ischemia. Complications were classified according to the Clavien–Dindo classification (Table 1) [13]. Only intraoperative bleeding and postoperative anemia that required a transfusion were classified as a complication (Clavien–Dindo 2), as pelvic surgery is per se associated with significant blood loss. The indication for transfusion during the surgery was made in consultation between anesthesia and trauma surgery staff based on established transfusion triggers, such as the Hb value measured in the BGA (blood gas analysis), hemodynamic parameters, and comorbidities [14-16]. In the case of postoperative anemia, the indication for transfusion was also made on the basis of established transfusion triggers, primarily the Hb value and comorbidities [17]. Routine transfusions were not performed at our clinic.

**Table 1:** Clavien-Dindo classification

Grade	Clavien-Dindo classification adapted from Dindo 2004 [13]
1	Any deviation from the normal postoperative course without the need for pharmacological treatment (excluding antiemetics, antipyretics, analgesics, diuretics, electrolytes and physiotherapy) or surgical (excluding wound infections opened at the bedside), endoscopic, and radiological interventions
2	Requiring pharmacological treatment with drugs other than those allowed for grade I complications, including blood transfusions and total parenteral nutrition
3	Requiring surgical, endoscopic or radiological intervention
	3a: Intervention not under general anesthesia
	3b: Intervention under general anesthesia
4	Life-threatening complication requiring intensive care/intensive care unit management
	4a: Single-organ dysfunction (including dialysis)
	4b: Multi-organ dysfunction
5	Death

Where available, the estimated blood loss during the surgery (mL) was collected from the anesthesia protocols and the decrease in hemoglobin level from preoperatively to the 1st postoperative day (g/dL) was determined from the laboratory results.

### Statistics

Statistical analysis was performed using Microsoft Excel (Microsoft Corp, Redmond, WA, USA) and R version 4.5.1 (R Foundation for Statistical Computing, Vienna, Austria) using the package ‘fmsb’. As this is a primarily descriptive and non-comparative case series, neither hypothesis testing nor sample size calculation was performed. Sex, indications for implant removal, surgical approaches used, localization/type of implants removed, number of patients with complications, and number of single complications were expressed as absolute and relative frequencies. Age, estimated blood loss during surgery, and decrease in hemoglobin level were expressed as mean with standard deviation (SD). To investigate whether the indication for implant removal, the surgical approach used, or the localization/type of implant has an influence on the complication rate, the subgroups were analyzed using risk differences (RDs) with 95% confidence interval (CI).

### Ethics, registration, data sharing plan, funding, and disclosures

The study was approved by the local ethics committee of the Hamburg Medical Chamber (2024-300544-WF) and the study was performed in accordance with the ethical standards of the 1964 Declaration of Helsinki. Registration of the study was completed in the German Clinical Trial Register (DRKS00037583). The data that support the findings of this study is available from the corresponding author upon reasonable request. There was no funding for this study. The authors declare no conflicts of interest. Complete disclosure of interest forms according to ICMJE are available on the article page, doi: 10.2340/17453674.2026.45294

## Results

The study comprises 141 patients who underwent implant removal from the pelvis. In 11 patients, the implant removal was conducted using more than 1 approach, resulting in 154 procedures that were analyzed (see Figure 1). The study cohort consisted of 112 (79%) male patients and 29 (21%) female patients (Table 2). The mean age was 49.5 (SD 14.7) years. The indications for removal, approaches used, and type/localization of plates removed are summarized in Table 2.

**Table 2:** Demographic and baseline clinical data of the study cohort. Values are count (%) or as specified

Factor	Study cohort
Age, mean (SD)	49.5 (14.7)
Sex (n = 141 patients)	
Male	112 (79)
Female	29 (21)
Indication for implant removal (n = 154)	
Infection	57 (37)
Interfering material	56 (36)
Removal for THA	34 (22)
Pseudarthrosis/re-osteosynthesis	7 (5.0)
Approach used for removal (n = 154)	
Kocher-Langenbeck	68 (44)
Modified Stoppa/AIP	49 (32)
Ilioinguinal	37 (24)
Type of plate removed (n = 154)	
Symphyseal plate	42 (27)
Anterior acetabulum and pelvic ring	45 (29)
Posterior acetabulum and pelvic ring	67 (44)

### Complications

During 53 surgeries (34%) at least 1 perioperative complication occurred. 72 single complications were identified, which are listed in Table 3. The most common complications were intraoperative bleeding requiring transfusion (hereafter “intraoperative bleeding”) (17/154; 11%), postoperative anemia requiring transfusion (hereafter “postoperative anemia”) (12/154; 7.8%), and vascular injuries (9/154; 5.8%). Patients who had intraoperative bleeding had an estimated blood loss of 2,321 (SD 1,722) mL (n = 15) compared with 1,111 (SD 1,035) mL in 89 patients without this complication. The average decrease in hemoglobin level from preoperatively to the 1st postoperative day for patients with postoperative anemia was 3.6 (SD 1.3) g/dL compared with 3.0 (SD 1.6) g/dL in 141 patients without this complication. Of all complications observed, 14 (9.1%) led to revisions: 6 due to hematoma, 7 for infection, and 1 for wound healing disorder.

**Table 3:** Complication risk following pelvic implant removal and frequency of individual complications with classification according to Clavien-Dindo (n = 154)

Complications with CD during pelvic implant removals	
Operations with complication	53 (34%)
Number of complications in total	72
Intraoperative bleeding with transfusion (CD 2)	17
Postoperative anemia with transfusion (CD 2)	12
Vascular injury (CD 3)	9
Infection (1 x CD 2 and 7 x CD 3)	8
Hematoma (CD 3)	6
Sciatic nerve involvement (CD 1)	4
LFCN involvement (CD 1)	3
Femoral nerve involvement (CD 1)	3
Urinary bladder injury (CD 3)	3
Wound healing disorder (2 x CD 1 and 1 x CD 3)	3
Deep vein thrombosis (CD 2)	3
Intraoperative myocardial ischemia (CD 4)	1

CD: Clavien-Dindo classification.

Subgroups were analyzed regarding the complication rates, the estimated blood loss during surgery, and the decrease in hemoglobin level depending on the indication for removal, the surgical approach, and the localization/type of implant (Table 4). Removal due to infection had a higher complication rate (44%) than pseudarthrosis/re-osteosynthesis (43%), interfering material (30%), or removal for THA (24%). Vascular injuries and intraoperative bleeding were predominantly observed during implant removal due to infection, resulting in the highest blood loss and decrease in hemoglobin level. When comparing removals for one-stage THA with planned removals/interfering material, implant removals with simultaneous THA had a higher risk of intraoperative bleeding (4/34; 12% vs 1/56; 1.8%), postoperative anemia (4/34; 12% vs 1/56; 1.8%) and higher estimated blood loss (1,346 [SD 1,114] mL vs 824 [SD 906] mL). Interestingly, all urinary bladder injuries in this study occurred during elective removal of interfering implants.

**Table 4:** Complication rates, estimated blood loss during surgery and decrease in hemoglobin level on the 1st postoperative day differentiated by indication for implant removal, surgical approach used, and type of plate removed in 154 surgeries

Factor	n	Surgeries with a complication n (%)	n	Estimated blood loss during surgery, mL mean (SD)	n	Decrease in hemoglobin level, g/dL mean (SD)
Indication for implant removal						
Infection	57	25 (44)	47	1,594 (1,419)	57	2.9 (1.6)
Interfering material/planned removal	56	17 (30)	28	824 (906)	55	2.6 (1.3)
Removal for one-stage THA	34	8 (24)	23	1,346 (1,114)	34	4.0 (1.4)
Pseudarthrosis/re-osteosynthesis	7	3 (43)	6	783 (317)	7	2.7 (1.5)
Approach used for removal						
Kocher-Langenbeck	68	21 (31)	51	1,415 (1,279)	67	3.7 (1.5)
Modified Stoppa/AIP	49	13 (27)	23	688 (720)	49	2.1 (1.4)
Ilioinguinal	37	19 (51)	30	1,522 (1,321)	37	3.0 (1.2)
Type of plate removed						
Symphyseal plate	42	11 (26)	18	518 (606)	42	2.1 (1.3)
Anterior pelvic ring/acetabulum	45	22 (49)	36	1,662 (1,527)	45	3.0 (1.4)
Posterior pelvic ring/acetabulum	67	20 (30)	50	1,290 (1,021)	66	3.6 (1.5)

n = number of procedures, SD = standard deviation.

The ilioinguinal approach exhibited a higher complication rate (19/37; 51%) than the Kocher-Langenbeck approach (21/68; 31%) and the modified Stoppa/AIP approach (13/49; 27%). Implant removal from the anterior pelvic ring/acetabulum was associated with a higher complication rate (22/45; 49%) than implant removal from the posterior acetabulum and pelvis (20/67; 30%) and removal of symphyseal plates (11/42; 26%). Injuries of the urinary bladder and vascular injuries were limited to anterior approaches (ilioinguinal approach and modified Stoppa/AIP approach) and to removals of symphyseal plates and implant removals from the anterior pelvic ring/acetabulum. Vascular injuries (RD 15%, CI 1–29) and intraoperative bleeding (RD 20%, CI 6–33) were observed significantly more frequently using the ilioinguinal approach (7/37; 19% and 8/37; 22%) than the modified Stoppa/AIP approach (2/49; 4.1% and 1/49; 2.0%). RDs for the overall complication rate depending on indication for removal, surgical approach, and the localization/type of implant are given in Tables 5, 6, and 7, respectively.

**Table 5:** Complication rate risk differences (RD) regarding the indication for removal in percentages with 95% confidence interval (CI). RD is calculated for the left column compared with the top row

RD % (CI)	Infection	Interfering material	Removal for THA	Pseudarthrosis
Infection	X	14 (–4 to 31)	20 (1 to 40)	1 (–38 to 40)
Interfering material	–14 (–31 to 4)	X	7 (–12 to 25)	–13 (–51 to 26)
Removal for THA	–20 (–40 to –1)	–7 (–25 to 12)	X	–19 (–59 to 20)
Pseudarthrosis	–1 (–40 to 38)	13 (–26 to 51)	19 (–20 to 59)	X

**Table 6:** Complication rate risk differences (RD) regarding the approach used for removal in percentages with 95% confidence interval (CI). RD is calculated for the left column compared with the top row

RD % (CI)	Kocher-Langenbeck	Modified Stoppa/AIP	Ilioinguinal
Kocher-Langenbeck	X	4 (–12 to 21)	–20 (–40 to –1)
Modified Stoppa/AIP	–4 (–21 to 12)	X	–25 (–45 to –5)
Ilioinguinal	20 (1 to 40)	25 (5 to 45)	X

**Table 7:** Complication rate risk differences (RD) regarding the type/location of the material removed in percentages with 95% confidence interval (CI). RD is calculated for the left column compared with the top row

RD % (CI)	Symphyseal plate	Anterior pelvis	Posterior pelvis
Symphyseal plate	X	–23 (–42 to –3)	–4 (–21 to 14)
Anterior pelvis	23 (3 to 42)	X	19 (1 to 37)
Posterior pelvis	4 (–14 to 21)	–19 (–37 to –1)	X

## Discussion

This is the largest study analyzing the risk of complications following implant removal from the pelvis and can therefore make a significant contribution to the decision-making process when removing implants from the pelvis is considered. We aimed to evaluate the complication rates of pelvic implant removals with respect to the surgical approach, type of implant, and indication for removal. We found an overall complication rate of 34%. The complication rate was influenced by the indication for removal, the type/localization of the implant as well as the surgical approach used. In particular, the ilioinguinal approach, implants at the anterior pelvic ring/acetabulum, and infection as the indication for removal were found to have particularly high complication risks.

Stuby et al. reported a complication rate of pelvic implant removal of about 20% in their retrospective case series of 80 pelvic ring fractures [18]. However, their analysis also included minimally invasive implant removals, such as removal of supraacetabular external fixators and SI screws, which may account for the lower risk profile. The complications reported were hematoma, bladder injuries, and remaining material. Blood loss or anemia requiring transfusion were not reported, which may also contribute to the different complication rates. Moreover, only 2 of their implant removals were due to infection. While implant removal surgeries due to patient request or interfering material allow for careful planning and evaluation of the indication, infection as indication does not. This might also explain the difference in overall complication rate between the 2 studies, as more than 1/3 of all our implant removals took place because of infection. The complication rate of over 40% highlights the complexity of implant removal in the case of infection, which may be explained by the altered anatomy and vulnerable tissue due to infection. No comparable data was found in the existing literature to date.

1 in 4 who had removal of pelvic implants for THA developed a complication in our cohort. In contrast, La Camera et al. found an overall complication rate of only 14%; however, they primarily described THA-related complications, not including blood loss or anemia requiring transfusion, and the rate of revisions. They reported postoperative transfusion in 18% of patients undergoing single-stage hardware removal and THA, which is comparable to the rate of 12% for both intraoperative and postoperative transfusion in our case series [19]. Although material removals due to infection were associated with the highest risk of intraoperative blood loss in this case series, cases involving removals for one-stage THA showed a markedly higher risk of postoperative anemia and higher estimated blood loss than planned removals/removals for interfering materials. This can be explained, at least in part, by the increased invasiveness and additional bone preparation for the THA performed at the same time. However, we believe that the osteosynthesis material removal during simultaneous THA significantly contributes to the risk of complications, as the preparation of the materials that may have been in place for decades is often difficult, invasive, and prone to complications.

In a retrospective study the overall complication rate (not including intraoperative bleeding or postoperative anemia requiring transfusion) was not higher for implant removal from the pelvis compared with implant removal from other locations, e.g., the extremities. Nevertheless, complications related to implant removal from the pelvis were described as far more severe and the risk for revisions due to complications at the pelvis was significantly higher than for implant removal at the extremities [20].

We observed a considerably higher complication risk when the ilioinguinal approach was used for implant removal compared with the Kocher-Langenbeck approach or the modified Stoppa/AIP approach. This is in line with the studies reporting on complication risks during primary stabilization depending on the surgical approach used. In a systematic review and meta-analysis, the modified Stoppa (AIP) approach showed a significantly lower blood loss, complication rate, and risk of postoperative infections than the ilioinguinal approach for the surgical fixation of acetabular fractures [21]. After the ilioinguinal approach, neuropraxia of the femoral cutaneous nerve and the femoral nerve is described in about 13% and 4%, respectively, which corresponds to our findings to some extent [22]. Our rate of intraoperative sciatic nerve injury is similar to the 5% rate described by Hakeem et al. [23]. Vascular injuries were reported in about 6% of cases with an ilioinguinal approach and in about 3% of cases with a modified Stoppa approach for primary fracture fixation [21]. In contrast, we identified vascular injuries in 4% of cases via the Stoppa/AIP approach but in 19% via the ilioinguinal approach, which may indicate the more challenging conditions when revision surgery like implant removal is performed. Formation of scar tissue and ossifications and thus an altered und more vulnerable anatomy makes implant removals more demanding and more prone to complications. This is further supported by the elevated overall complication rates observed in our study cohort: 51% for the ilioinguinal approach, 27% for the Stoppa/AIP approach, and 31% for the Kocher-Langenbeck approach. In contrast, a systematic review found lower complication rates of 7%, 17%, and 5% for the respective approaches for primary fixation of pelvic ring and acetabular fractures. However, comparability is limited here, as they did not report intraoperative bleeding and anemia requiring transfusion [24].

The indication for pelvic implant removal must be carefully considered. Except for absolute indications for implant removal from the pelvis, such as external fixators, in the case of infection or malpositioned implants, other reasons for implant removal are controversial [25-27]. Particularly in the case of explicit patient request and in case of pro forma material removal when the fracture is healed, the indication for implant removal should be considered critically in view of the high complication rate observed in this study [18,28]. It is also important to inform the patient in detail about the high risk of complications to enable an educated patient decision [7]. The high risk of complications must by justified by a thorough assessment of the patient’s medical history and examination to rule out other causes for the complaints such as degenerative joint disease before a decision on implant removal is made [18].

### Limitations

Due to the retrospective design and the lack of standardized postoperative follow-up, minor or late complications that were treated externally or not documented in the patient file could not be considered. This may lead to an underreporting of complications. Being a single-center study, information bias cannot be ruled out. An also popular anterior approach nowadays, the pararectus approach, was not used for hardware removal in this cohort and therefore could not be compared with the Stoppa approach in this case series. Additionally, due to the specialization and catchment area of the study center, the case series includes several highly complex revision cases in which a particularly high complication rate is to be expected. This may limit the representativeness of the study cohort. Furthermore, the decision for implant removal and surgical approach was not standardized but left to the individual decision of the treating surgeon and the patient. Nevertheless, the comparability of the study participants was increased by exclusion of hardware removal with a differing complication risk profile, such as isolated minimally invasive removal of SI screws and external fixators. A multivariable analysis including comorbidities, timing of implant removal, and other preoperative risk factors, such as use of anticoagulation or BMI, was not possible due to incomplete and non-standardized reporting of this clinical data in the patient files. As a result, potential bias due to unobserved confounders, e.g., patient- and surgery-related factors, cannot be ruled out.

## Conclusion

This study demonstrated a significant overall complication rate, in 1 of 3 patients following removal of osteosynthesis materials from the pelvic ring and acetabulum. Cases with deep infections, where implants at the anterior pelvic ring/acetabulum are removed and where the ilioinguinal approach is used, are associated with an increased complication risk.

*In perspective*, a liberal indication for hardware removal should not be given in cases of potentially interfering material, loosened implants, or material breakage of symphyseal plates due to the high risk of complications. Loosened or broken material alone does not justify hardware removals, particularly in asymptomatic patients. The findings of this study should be used to support clinical decision-making and to inform patients regarding the potential risks of hardware removal.

## Notes

Conceptualization: NH, MH. Data curation: TCS, NH. Formal analysis: TCS, NH. Investigation: TCS, NH. Methodology: TCS, NH, CG, MH. Project administration: KHF, MH. Supervision: CG, KHF, MH. Writing—original draft: TCS. Writing—review and editing: NH, MH.

Handling co-editors: Cecilia Rogmark and Robin Christensen

*Acta* thanks Madelene Albrektsson and Morten Schultz Larsen for help with peer review of this manuscript.
